# Intraoperative Guidance Using Hyperspectral Imaging: A Review for Surgeons

**DOI:** 10.3390/diagnostics11112066

**Published:** 2021-11-08

**Authors:** Manuel Barberio, Sara Benedicenti, Margherita Pizzicannella, Eric Felli, Toby Collins, Boris Jansen-Winkeln, Jacques Marescaux, Massimo Giuseppe Viola, Michele Diana

**Affiliations:** 1 Institute for Research against Digestive Cancer (IRCAD), 67091 Strasbourg, France; toby.collins@ircad.fr (T.C.); jacques.marescaux@ircad.fr (J.M.); michele.diana@ircad.fr (M.D.); 2 General Surgery Department, Ospedale Card. G. Panico, 73039 Tricase, Italy; katutopia.l@gmail.com (S.B.); marghe.pizzicannella@gmail.com (M.P.); masgiuviola@yahoo.it (M.G.V.); 3 Department of Visceral Surgery and Medicine, Inselspital, Bern University Hospital, University of Bern, 3008 Bern, Switzerland; eric.felli@dbmr.unibe.ch; 4 Department for BioMedical Research, Visceral Surgery and Medicine, University of Bern, 3008 Bern, Switzerland; 5 Department of General Surgery, St. Georg Hospital, 04129 Leipzig, Germany; jansen-winkeln@gmx.de; 6 ICube Laboratory, Photonics Instrumentation for Health, University of Strasbourg, 67400 Strasbourg, France

**Keywords:** hyperspectral imaging, optical imaging, image-guided surgery, intraoperative guidance, intraoperative imaging, precision surgery

## Abstract

Hyperspectral imaging (HSI) is a novel optical imaging modality, which has recently found diverse applications in the medical field. HSI is a hybrid imaging modality, combining a digital photographic camera with a spectrographic unit, and it allows for a contactless and non-destructive biochemical analysis of living tissue. HSI provides quantitative and qualitative information of the tissue composition at molecular level in a contrast-free manner, hence making it possible to objectively discriminate between different tissue types and between healthy and pathological tissue. Over the last two decades, HSI has been increasingly used in the medical field, and only recently it has found an application in the operating room. In the last few years, several research groups have used this imaging modality as an intraoperative guidance tool within different surgical disciplines. Despite its great potential, HSI still remains far from being routinely used in the daily surgical practice, since it is still largely unknown to most of the surgical community. The aim of this study is to provide clinical surgeons with an overview of the capabilities, current limitations, and future directions of HSI for intraoperative guidance.

## 1. Introduction

Over the last few decades, the impressive advances in the fields of computer science and imaging technologies have increased the machine/clinician synergy, bringing precision medicine into the current clinical practice [1]. In particular, the constant development of new devices and minimally invasive platforms together with the implementation of advanced imaging technologies has led to an epochal change within surgical disciplines. In fact, while the indications for minimally invasive surgical procedures are progressively extended towards more complex pathologies, traditional diagnostic disciplines such as radiology or endoscopy are developing an increasing portfolio of minimally invasive treatment options. In this context, the intraoperative use of imaging technologies which can augment the human sight are fundamental to increase the accuracy and precision of surgery. The ideal intraoperative imaging modality should be safe and user-friendly to smoothly fit within the operating workflow [2,3]. Additionally, it should provide reproducible and quantitative results without the need for an exogenous labelling agent [4], enriching the surgeon with additional useful information and assistance in the decision-making process.

In this view, hyperspectral imaging (HSI) displays most of the features of the ideal intraoperative imaging technology, as it can provide a qualitative and quantitative snapshot of the biological tissue’s chemical properties in a non-invasive, radiation-free, label-free, and user-friendly manner. HSI is included within the optical imaging domain, and it results from the combination of a digital camera with a spectrometer. The interaction of light with the target object generates specific signatures or fingerprints across the electromagnetic spectrum, which are detected with the spectrometric unit. The resulting dataset is a spatially and spectrally resolved three-dimensional set of information, called a hypercube (spatial coordinates: x, y; spectral coordinate: z). Given its ability to rapidly discriminate and quantify chemical composition over large areas, HSI has been successfully used within the fields of remote sensing [5], food quality control [6], vegetation and water resource control [7], forensic medicine [8], recycling industry [9], analysis/restoration of old paintings or manuscripts [10].

HSI can distinguish the biochemical composition of healthy and pathological biological tissue [11] in a non-invasive fashion, and for this reason, it has been increasingly used in the medical field [12,13]. Previously, other authors have written comprehensive overviews concerning the application of HSI in gastroenterology [14], surgery [15] or surgical clinical studies [16]. However, those previous works have been meant for biomedical engineers/scientists [14,15] and contain a high degree of theoretical and technical details, and they are rather difficult to digest for the average surgeon. Alternatively, overview articles have been written before 2018 [16], and starting from that period, several experimental and clinical works reporting interesting novel intraoperative applications using HSI have been published.

The aim of this study is to provide a snapshot for the surgical audience of the diverse applications of hyperspectral imaging as an intraoperative imaging tool, supported by human and experimental studies, and to discuss its current limitations and possible future directions.

## 2. Theoretical Overview

This article is written for a clinical audience. As a result, the technical details regarding biophotonics, hardware engineering, data extraction and processing will be only concisely mentioned since they go beyond the purpose of this manuscript. For a more comprehensive technical and theoretical description of the principles behind HSI, we refer the interested reader to previous works [12,13,14,15].

Biophotonics, from the ancient Greek bios (βίος) meaning life, and fos (φῶς) meaning light, is the science studying the interactions between biological tissue and light. Once light enters living tissue, it induces several phenomena (mainly scattering, adsorption, reflection), which strictly depend on the chemical composition of the specific tissue. For this reason, each tissue type presents a characteristic optical pattern, named spectral signature or spectral fingerprint, which can be used to differentiate it from another tissue type. Similarly, the spectral signatures allow to discriminate pathological (e.g., cancerous, ischemic or burned tissue) from healthy tissue [11].

### 2.1. The Electromagnetic Spectrum and the Hypercube

**Figure 1:**
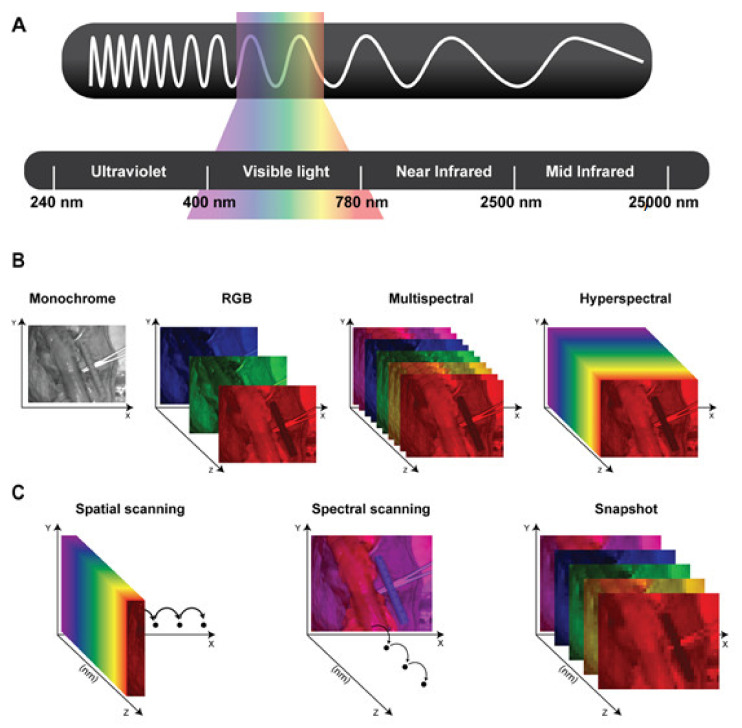
(**A**) Schematic representation of the electromagnetic spectrum’s wavelengths: Ultraviolet (240–400 nanometers), Visible light (400–780 nanometers), Near-Infrared (780–2500 nanometers, Mid- Infrared (2500–25000 nanometers). (**B**) Representation of the different datasets generated using: monochrome images; color or RGB (Red, Green, and Blue) images; multispectral and hyperspectral imaging. (**C**) Schematic representing the 3 different types of hyperspectral imaging devices. Spatial scanning: acquiring the spectral information as a whole and progressively scanning the spatial information. Spectral scanning: acquiring the spatial information as a whole and scanning the spectral one. Snapshot: simultaneously acquiring spectral and spatial information but providing a lower spatial and spectral resolution than previously mentioned hyperspectral imagers.

The human eye only detects a narrow portion of the electromagnetic (EM) spectrum. In fact, human sight can only perceive light in the visible (VIS) range (400–780 nm) (Figure 1A). Light in the VIS range exhibits only minimal tissue penetration (1–2 mm), while in the near-infrared (NIR) range (780–2500 nm) it can pass up to several millimeters through tissue, hence having a higher diagnostic value than VIS [12].

HSI is a hybrid imaging modality which combines digital photograph and spectrometry, generating a three-dimensional dataset, namely the hypercube. This is composed of a stack of two-dimensional images (spatial coordinates) across a wide and generally contiguous range of the EM spectrum (spectral coordinate), hence augmenting the human vision far beyond its natural capabilities (Figure 1B). HSI is a spatially resolved spectroscopy, allowing us to measure the spectral signature at every pixel. This imaging modality covers large contiguous portions of the EM spectrum, typically from UV to NIR, and possesses more spectral bands (up to several hundreds) than multispectral imaging (which has usually up to 12). As a result, HSI exhibits a much larger amount of information and consequently possesses a greater diagnostic potential than multispectral imaging (Figure 1B). Each pixel of the hypercube contains a spectral curve. For this reason, the hypercube yields a massive amount of information. Depending on the diagnostic purpose, different parts of the spectral signature might contain the relevant discriminative information. For this reason, data extraction and processing are fundamental and has complex steps, which are strictly dependent on the specific application. As a result, the assistance of advanced machine learning (ML) algorithms is required, and the great advances made with hyperspectral data extraction in the field of remote sensing [17], gradually using more sophisticated ML strategies, such as deep learning, are progressively used and adapted into medical HSI.

### 2.2. Types of Hyperspectral Imaging Hardware

Hyperspectral imagers are categorized into two main groups, according to the data acquisition mechanism, namely scanning devices (divided into spatial and spectral scanning) and snapshot devices (Figure 1C).

Spatial scanning devices acquire the whole spectral information and progressively scan the spatial information. They are either point scanning (wiskbroom), if they scan the spatial information pixel by pixel, or line scanning (pushbroom) if they scan the spatial information line by line. This kind of device typically shows a high spectral resolution, while the spatial resolution is limited by the number of lines or pixels of the image. However, the tradeoff between the spatial and spectral information is often acceptable. Consequently, they are widely used in the medical field [12]. However, the spatial scanning mechanisms are often bulky and require complex hardware, making their miniaturization [18] challenging [19,20]. Another main disadvantage of spatial scanning systems is that the acquisition is not made in real time and it is limited by the speed of the spatial scanning process that may take several seconds. As a result, the accuracy of spatial scanning devices is limited during the acquisition of moving targets, and they tend to produce motion artefacts due to breathing or heart beating, and most importantly, they provide static images.

Spectral scanning devices acquire the spatial information as a whole and they possess a tunable optical element, which allows to image the target using different wavelengths, depending on their spectral coverage. The optical element can be controlled either manually, or automatically as in the case of the Liquid Crystal Tunable Filter (LCTF) or the Acousto-Optic Tunable Filter (AOTF) devices. Considering that the hardware of those cameras is simpler than spatial scanning systems, they can be easily attached to existing optical devices, such as microscopes [21], laparoscopes [22], and flexible endoscopes [23]. However, they are limited by a considerably lower spectral resolution than spatial scanning devices. Of note, they are even more sensitive than spatial scanning cameras to motion artefacts since their spatial information is acquired repeatedly for each spectral wavelength. As a result, this kind of spectral imager also does not allow for real-time imaging.

Snapshot devices can quickly and simultaneously acquire spectral and spatial information. They allow for real-time imaging at the cost of spectral and spatial resolution, which are lower than spectral and spatial scanning devices. Ideally, imaging devices used during surgical procedures must display the spatial information with a high degree of accuracy. This is fundamental to localize the supplementary information onto the surgical field, and as such, it is an advanced imaging modality disclosed to the surgeon. Given their poor spatial resolution, snapshot cameras have been rarely used intraoperatively [14].

In most of the previously published experimental and human studies, the HSI systems used were custom-made. However, as comprehensively described elsewhere [15], there are only a few commercially available hyperspectral systems on the market (and many of the available spectral imagers are multispectral) and still fewer are designed or approved for clinical applications.

## 3. Hyperspectral Imaging as an Intraoperative Imaging Tool

Hyperspectral imaging is an emerging technology in medicine, particularly in the surgical field. For this reason, the number of studies involving HSI as an intraoperative guidance tool is limited. As previously mentioned, the existing reviews are thoroughly written, but they emphasize biomedical or technical aspects, which can be too intricate for surgeons and inevitably go beyond their needs. The rationale behind the current study is to provide surgeons with an overview of the possibilities and perspectives that HSI offers as an intraoperative imaging tool. The literature for our review was retrieved from PubMed and Google Scholar. Search terms included hyperspectral imaging (excluding multispectral), surgery, intraoperative. We included human and animal studies involving only large animal models, due to their straightforward translatability into clinical practice, in comparison to small animal studies. Ex vivo studies were included only if the HSI acquisition was performed in the operating room. Case reports or studies in a language other than English were excluded.

Intraoperative HSI has been mainly applied to two large areas, divided into subcategories (Table 1). The first is “tissue recognition” composed of (i) cancer recognition; (ii) anatomical structures recognition; and (iii) thermal ablation efficacy recognition. The second is “perfusion assessment” within: (i) colorectal surgery; (ii) upper gastrointestinal surgery; (iii) hepatopancreaticobiliary surgery; (iv) reconstructive surgery; (v) urology; and (vi) neurosurgery.

## 4. Tissue Recognition

During oncologic surgical procedures, it is fundamental to be radical, which means the complete removal of neoplastic tissue. Currently, a radical oncologic resection is achieved in minimally invasive procedures, exclusively from the surgeon’s visual assessment, since haptic feedback is not available. For this reason, the pathologist’s assistance is often required, and an intraoperative frozen section is mandatory to objectively evaluate the resection margin, at the cost of a greater operating time. Preliminary studies reporting the intraoperative visual enhancement of cancerous tissue are available and showed promising results in terms of detection rate accuracy. However, this typically calls for the use of unlabeled [59] or labeled [60] exogenous agents, which might be potentially responsible for adverse reactions and are still “off label” for this kind of application.

Diversely, it is highly desirable to intraoperatively recognize anatomical structures (such as nerves, ureters, parathyroid glands, common bile duct, etc.) to spare them during the demolition phase and preclude any accidental injury, which could cause important functional deficits which negatively affect patient quality of life. Again, this process is currently strictly dependent on the surgeon’s visual capabilities and anatomical knowledge and currently there are no universally accepted methods to recognize noble anatomical structures intraoperatively. However, several experimental methods using optical imaging modalities, but still requiring an injection of exogenous contrast agents [61,62,63] or the use of fluorescent devices [64,65,66], have been put forward to highlight key anatomical structures within human or experimental models.

On the other hand, HSI has the potential to efficiently discriminate between different tissue types in a contrast-free and non-destructive manner. Theoretically, it is an ideal intraoperative guidance tool which allows to identify either pathological tissue or essential anatomical structures on the surgical scene.

### 4.1. Cancer Recognition

As previously mentioned, the hypercube is a large dataset, and information that is required to discriminate between different tissue types must be extracted. As a result, machine learning algorithms are used to perform this task automatically. Ideally, to assist the surgeon, an HSI imaging system should automatically detect the different tissue types immediately or shortly after the acquisition and provide the operator with simple visual feedback. Deep learning algorithms, in particular neural networks, can promptly differentiate between different tissues, based on their spectral characteristics, either completely automatically (unsupervised learning) or after learning from previously annotated images (supervised learning). Although many previous works have focused on the detection of cancerous tissue using HSI coupled with various data processing algorithms [67], most of them were performed ex vivo (in the histopathological lab) or in vivo, but using small animal models. The HELICoiD (HypErspectraL Imaging CancerDetection) is a multidisciplinary group of scientists focused on building a hyperspectral imager especially suited for the in vivo detection of brain tumors, which has been successfully tested in human cases [24]. Successively, the same group used a deep learning classifier, which achieved an acceptable differentiation between cancerous and normal parenchymal tissue (sensitivity 88%, specificity 100%), outperforming simpler machine learning algorithms [25]. Interestingly, in another study, the authors discovered the relevant EM wavelength ranges required to discriminate brain tumors using in vivo acquisitions performed with their custom-made camera. They improved the tumor detection accuracy of machine learning algorithms, by using only those relevant bands [26]. This approach was differently tailored according to each specific diagnostic query represents, an important step towards instantaneous automatic recognition. In fact, by limiting image acquisition specifically to the discriminative wavelengths and by creating custom-made algorithms, which analyze only preselected parts of the hypercube, the time between imaging and information output is enormously reduced.

As far as recognition of gastrointestinal tumor is concerned, an interesting study using a modified flexible endoscope to recognize colorectal tumors in vivo in human subjects has been published [23]. However, this study involved HSI as a preoperative diagnostic tool and not as an intraoperative tool. Most works on this subject have been performed ex vivo, mainly after specimen processing in the pathology department.

In a recent pilot study conducted on fresh specimens of patients undergoing colorectal resections [27] (acquisition still in the operating room, immediately after removing the specimen from the body), using HSI combined with artificial neural networks (specifically multilayer perceptron), the authors were able to discriminate colorectal cancer from healthy tissue with a good degree of precision (sensitivity 86%, specificity 95%). Additionally, relevant spectral differences, reflecting variations in concentrations in terms of oxygenated hemoglobin or water content, were encountered. Interestingly, those variations were related to important oncologic variables, such as local tumor extension or the presence of previous neoadjuvant chemotherapy. Although this was a relatively small (54 patients) and monocentric study, the finding of spectral differences depending on tumor extension or neoadjuvant chemotherapy opens new clinical insights. In fact, this shows that HSI might soon potentially assess the efficacy of a neoadjuvant treatment or a complete local tumor remission after medical treatment, for instance. This would open new therapeutical strategies, possibly involving multidisciplinary patient-tailored treatments. However, it must be emphasized that HSI is a surface technique, therefore only superficial parts of the cancer tissue can be analyzed using this technology. Additionally, although encouraging, those are merely preliminary results that need to be confirmed on larger patient numbers in order to draw meaningful conclusions.

Recently, a French–German collaborative group using fresh specimens of 12 patients with colorectal cancer and 10 with esophageal cancer, were able to achieve an acceptable automating recognition accuracy by using several machine learning supervised methods (Receiver Operator Curve Area-Under-Curve: ROC-AUC of 0.92 for colorectal and of 0.93 for esophagogastric cancer, respectively) [28]. Interestingly, the authors noticed a significant improvement in the automatic classification by combining both cancer types within the learning datasets. Although this study involved a rather small number of patients (namely 22), the interesting novel approach of combinations for the algorithm training phase of different histological tumor types proved able to improve the automatic classification accuracy. This method could be useful when dealing with limited datasets (e.g., small tumors, small patient cohorts, rare tumor types) in order to maximize the machine learning algorithm prediction efficacy.

Research in the field of brain cancer detection using HSI has made considerable improvements towards the intraoperative automatic recognition of pathological tissue, due to the introduction of complex deep learning algorithms and the discovery of tumor-specific spectral bands. This certainly originates in the existence of a large multicentric project, such as HELICoiD, which has concentrated the joint efforts of researchers from different disciplines. However, it must be mentioned that most of the oncologic neurosurgical procedures are performed in an open surgical setting by means of craniotomy. For this reason, intraoperative HSI imaging using bulky hardware is technically feasible and does not represent a burden for the surgical workflow. On the contrary, in digestive surgery, the oncologic procedures are increasingly performed using minimally invasive platforms (such as laparoscopy, robotics). Currently, no reliable HSI system suitable for minimally invasive gastrointestinal surgery is available because miniaturization of the HSI unit often goes at the cost of spectral and spatial resolutions. Another reason for the scarce advances in the field of intraoperative HSI recognition of gastrointestinal tumors is that those are mostly endoluminal neoplasia, and the surgeon has an extraluminal perspective during the procedure. However, as HSI can also detect light in the NIR spectrum, which has a deeper tissue penetration, it theoretically also has the potential to detect cancerous tissue within deeper tissue layers from the extraluminal side. Currently, our group is putting considerable effort into a research project aimed to detect endoluminal gastrointestinal tumors from the serosal side, and preliminary results are encouraging.

### 4.2. Recognition of Anatomical Structures

In a pioneer study, Zuzak et al. [29] built a laparoscopic HSI camera prototype, using a commercially available laparoscopic camera customized with a spectral scanning (LCTF) HSI system. The authors used this imager during a porcine surgical procedure and could successfully identify the specific spectral characteristics of the portal vein, hepatic artery, and common bile duct.

Another group focused their work on the recognition of porcine facial nerves and ureters in vivo [30]. Interestingly, they sought the best spectral bands to visually enhance the desired structures (nerve or ureter) intraoperatively on RGB pictures.

Wisotzky et al. [31] presented a different approach. They built a custom-made HSI analyzer with a light source capable of emitting monochromatic light in 16 different wavelengths and used it successfully in vivo in 8 human subjects during different surgical procedures (such as mastoidectomy, parotidectomy, and neck dissection). The authors then successfully analyzed the spectral characteristics of several human tissue types in the different wavelengths using a sophisticated post-processing set-up. This work was propaedeutic to future research aimed to identify human tissue types using the same custom-made camera combined with further ML algorithms.

**Figure 2:**
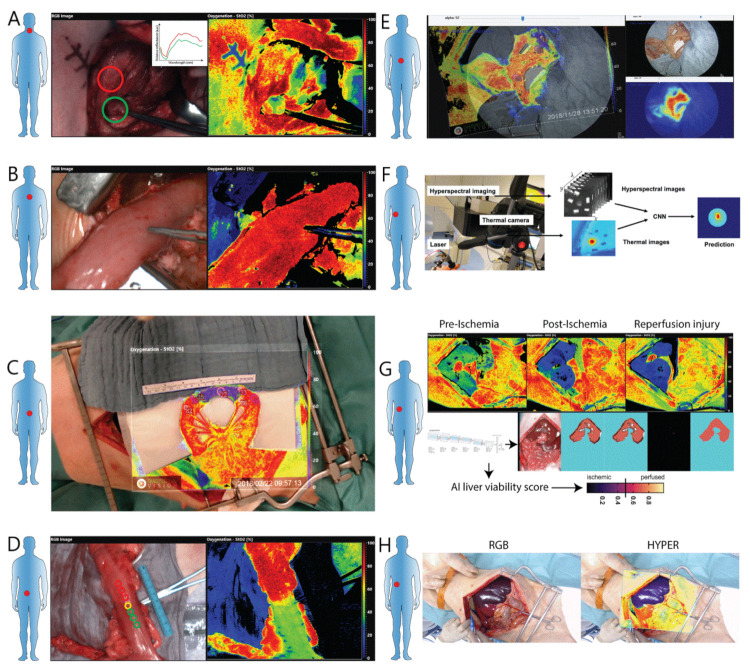
(**A**) Intraoperative HSI picture acquired during human thyroidectomy. In the red circle, thyroid tissue is highlighted, while the parathyroid gland is highlighted in the green one. The false-color image aside shows the perfusion quantification of the oxygen saturation (StO_2_); the parathyroid gland, highlighted with the forceps has less StO_2_ than the surrounding thyroid tissue. (**B**) Intraoperative HSI acquisition displaying the StO_2_ assessment of a gastric conduit during human esophagectomy. (**C**) A porcine small bowel ischemia model, in which the mesentery is divided into the middle is shown. The picture shows an example of the HYPER (HYperspectral Enhanced Reality) software, which overlays the HSI perfusion cartography onto the real-time video. This approach facilitates intraoperative navigation by means of HSI. (**D**) HSI acquisition made during human left colonic resection. The proximal resection margin is assessed with HSI. The surgeon set the forceps at the visually determined proximal resection line, obtained after interrupting the organ’s vascular supply. In the HSI-based StO_2_ quantification aside, it is visible that the visually determined resection line falls into the marginally perfused area (green) and not into the well-perfused area (red). This example shows that the visual assessment of bowel perfusion is often inaccurate. (**E**) Porcine pancreas partial ischemia model visible in the color picture (top left) with representation of the fluorescence angiography quantification (top right) and HSI-based StO_2_ quantification (bottom picture), showing a well-demarcated ischemic area (in blue). (**F**) Experimental set-up of the porcine hepatic thermal ablation monitoring using HSI. The thermal camera and HSI camera are used to train CNNs to predict liver damage induced by laser ablation with a high degree of accuracy. (**G**) Automatic liver viability scoring with deep learning and hyperspectral imaging. CNNs are trained to automatically recognize liver tissue and its perfusion (ischemic 0–perfused 1). During the reperfusion phase, the score predicts the ischemia reperfusion injury and microvascular failure. (**H**) Hyperspectral enhanced reality (HYPER) for anatomical liver resections. HSI images are overlaid over the real-time video to guide liver resection. This approach helps the surgeon to intraoperatively distinguish the non-perfused from the perfused parenchyma in order to guide liver transection.

In a pilot study in 9 human patients undergoing endocrine neck procedures, Barberio et al. acquired intraoperative HSI images and could find the specific spectral features of thyroid and parathyroid glands [32]. This preliminary work was the precursor of a second one [33], in which a similar dataset was analyzed with a supervised classifier and automatic recognition of the parathyroid gland was achieved (Figure 2A) against the surrounding tissue types. However, the accuracy of the automatic detection was rather low to be acceptable within a clinical setting. For that reason, another study was performed [34] focusing on automatic tissue recognition in animals. During neck dissections in several pigs, in vivo HSI acquisitions were made, and different tissue classes (such as muscle, nerve, vessels, skin, etc.) were annotated. Several post-processing algorithms including convolutional neural networks (CNN) were used, and automatic multiclass tissue recognition was achieved with a high degree of precision.

Encouraged by the result of the former study, our group is currently conducting research into human subjects with the aim of intraoperatively achieving automatic multiclass tissue recognition, using advanced ML strategies.

### 4.3. Thermal Ablation Efficacy Recognition

Percutaneous thermal ablation is gaining importance as a minimally invasive procedure in patients with primary or secondary focal liver tumors unable to undergo surgery [68]. The locally applied thermal treatment causes irreversible damage to the cancerous cells. However, at the current stage, the efficacy of this treatment is exclusively assessed by monitoring the temperature, either with non-invasive magnetic resonance thermometry or with invasive temperature sensors. HSI, with its ability to assess the chemical composition of the biological tissue, has the potential to detect irreversible tissue alterations following thermal ablation. Using an open surgical set-up, researchers performed hepatic laser thermal ablations in an in vivo porcine model and by analyzing the liver’s surface with HSI, they assessed a 40% increase in the spectral curves of the ablated tissue at the methemoglobin peak [35]. In a further study with a similar set-up, researchers monitored the hepatic thermal ablation with HSI and an infrared thermal camera simultaneously [36]. Subsequently, the spectral integrals corresponding to thermal-sensitive chromophores were extracted, and preliminary indicators of the thermal damage were proposed, successfully correlating to the thermal information (Figure 2F).

In a further (unpublished) study, the same group used a similar experimental set-up and created a deep learning model for thermal damage assessment from HSI data. They could successfully correlate the model’s predictions with thermal data and to histopathological data, demonstrating that HSI is suitable to assess the efficacy of hepatic laser thermal ablations in combination with appropriate deep learning algorithms.

However, thermal ablative procedures are meant to be percutaneous interventions and all the abovementioned studies were proofs of the concept, performed in an open surgical setting. While studies were performed using a commercially available camera, approved for human use, this system is limited by its exclusive suitability for open surgery. This represents an important drawback for a prompt clinical translation of this kind of approach. Additionally, since HSI analyzed only the organs’ surface and thermal ablation is mostly reserved for deeper lesions, it is difficult to imagine a prompt clinical translation of this HSI application.

## 5. Perfusion Assessment

In general, in every surgical discipline, a poor blood supply impairs the correct healing of the tissue. However, clinical evaluation alone is insufficient to reliably assess perfusion intraoperatively [69]. In the past, several imaging modalities have been explored to quantify ischemia intraoperatively. Fluorescence angiography (FA), a modality using the injection of an exogenous fluorophore, typically indocyanine green (ICG), has found a wide acceptance within the surgical community [70]. However, FA lacks a quantitative metric, and it requires the use of a contrast agent, which can be occasionally related to adverse events [71].

On the other hand, as HSI provides a quantitative and qualitative evaluation of the tissue in a contrast-free manner, it has great potential as an intraoperative imaging technology. However, the lack of a video rate with acceptable spatial resolution and the absence of a stable HSI platform for minimally invasive surgery currently represent consistent drawbacks for its diffusion in clinical practice as an intraoperative imaging tool.

### 5.1. Perfusion Assessment in Colorectal Surgery

In the field of gastrointestinal surgery, an insufficient blood flow is a well-recognized cause of anastomotic leakage, which is the most feared complication following a digestive surgical procedure. FA has been widely used to intraoperatively measure blood supply during colorectal procedures [72]. However, due to the lack of a unitary quantitative metric, together with the heterogeneity of the existing trials, its usefulness in daily clinical practice remains controversial.

In a pioneer study, Akbari et al. [37] created a small bowel ischemia model in a pig and used a bulky custom-made HSI system covering a large spectral range. The authors could identify the key wavelengths, which allowed for the best differentiation between normal and ischemic tissue, demonstrating that HSI is suitable to detect bowel ischemia.

However, in the remarkable proof of concept of Akbari, a large HSI system with a poor spatial resolution was used, and such a system would be unsuitable for clinical intraoperative use. Inspired by the study of Akbari, our group conducted a set of studies with a compact spatial scanner HSI camera approved for human use [20]. This camera has a short acquisition time (<10 s) and provides a quantitative heatmap of several physiological parameters, such as tissue oxygen saturation (StO_2_), near-infrared perfusion index (NIR), tissue water index (TWI) as immediate output, thanks to the algorithms integrated in the software. However, despite the good spatial resolution and the quantification of perfusion parameters, as the camera is a push-broom system, it delivers static images only. Static images are difficult to handle intraoperatively and only allow for an approximate localization of the region of interest (ROI) on the surgical scene. As a result, by customizing the commercially available HSI system, with the addition of a high-resolution video camera, and thanks to an in-house software solution, the system could provide a live video, displaying the quantitative perfusion information obtained with HSI in augmented reality. The solution was named HYPerspectral-based Enhanced Reality (HYPER) [38] and it allowed accurate intraoperative quantification of bowel perfusion. The perfusion quantification accuracy was tested in a complex experimental set-up involving a small bowel ischemia model in 6 pigs, with an ischemia time ranging from 5 min to 6 h. HYPER’s perfusion quantification was validated through a good correlation with robust ischemia biomarkers and it allowed for the precise localization of the HSI information on the surgical scene (Figure 2C).

Currently, fluorescence angiography is facing a wide acceptance as an intraoperative perfusion assessment tool in digestive surgery within the surgical community [70]. However, the debate on its usefulness remains open since FA does not provide a quantification and its interpretation remains mainly subjective. As a result, several FA quantification algorithms, which are currently not universally accepted, have been introduced into clinical practice [73,74]. In a further study [75] using a similar experimental set-up as the one previously described to validate HYPER, the perfusion quantification accuracy of HYPER was compared with an already thoroughly validated FA quantification algorithm [74,76,77,78,79]. Interestingly, HYPER showed a better correlation with the ischemia biomarker and showed a specific suitability in identifying marginally perfused bowel areas. Despite the excellent accuracy of HYPER, this technology presents several drawbacks impairing its use in daily practice. First, the perfusion quantification is not performed in real time, since after acquisition the HSI information is overlaid onto the live video of the surgical scene, a process which takes approximately 30 s. For this reason, the user would need to reacquire the HSI image and then overlay it onto the real-time video, as desired, to refresh the perfusion assessment. Secondly, this kind of approach is subject to image misalignment during surgical manipulation. As a result, further development in terms of image deformation and tracking is required before attempting clinical translation of this approach.

However, HSI (without HYPER) has been used during human minimally invasive colorectal resections to assess intestinal perfusion prior to anastomosis formation (Figure 2D). In a first pilot study in 24 patients, the ability of HSI to detect ischemia was confirmed [39]. Additionally, it was observed that the clinically determined resection line was often misplaced in comparison to the one determined with HSI. This difference was so substantial in 5 patients that the resection line was moved as suggested by the HSI acquisition. In a second study [40], using a similar set-up as previously described, the ability to detect the transection line of HSI was tested against (non-quantitative) fluorescence angiography. The results indicated that the resection lines corresponded in both methods. However, since HSI is a contrast-free technique, it is safer than FA and it allows for repeated perfusion assessments, while FA is influenced by the background fluorescence within the tissue of previously injected ICG. In a further study, HSI was successfully used as a guidance tool during emergency bowel resections to quantitatively assess intestinal perfusion within a small series of patients with acute mesenteric ischemia [41].

All abovementioned clinical studies were pilot trials, designed to test the usability of this novel technology within the operative workflow during digestive surgery cases. HSI could successfully identify ischemic bowel segments and quantify bowel perfusion. However, larger studies with well-designed set-ups are required to understand the clinical value of HSI-based decision-making tools to assess the gastrointestinal tract’s perfusion. In this view, our group is currently recruiting patients for a multicentric clinical trial comparing HSI to quantitative fluorescence angiography during colorectal resections.

### 5.2. Perfusion Assessment in Upper Gastrointestinal Surgery

Similarly as for colorectal surgery, an excellent blood supply is essential to ensure the proper healing of upper gastrointestinal tract anastomoses. This is even more critical in the case of esophageal resection, a major surgical procedure associated with consistent morbidity and mortality [80] in particular, with a higher rate of anastomotic leaks than other gastrointestinal procedures [81]. The higher complication rate of esophagectomy is intrinsic to the esophageal reconstruction technique, which traditionally involves a stomach graft, namely the gastric conduit. The gastric conduit is obtained by performing a sleeve gastrectomy, and the resulting stomach graft is tenuously supplied by a main single vessel left in place, i.e., the right gastroepiploic artery.

The HYPER concept was tested during gastric conduit formation in pigs, using a similar set-up as in bowel ischemia experiments [42]. Additionally, in this study, confocal laser endomicroscopy (CLE), a high-resolution optical microscopy technique which can assess microcirculation [82,83], was used simultaneously. After gastric conduit formation, which implies the surgical removal of most of the gastric blood supply, perfusion was quantified with HYPER from the serosal side and with CLE from the mucosal side. Interestingly, both techniques could precisely measure gastric perfusion, correlating well with one another and with the ischemia biomarker. In a second experimental study, an innovative ischemic preconditioning technique, aimed to improve gastric circulation following gastric conduit formation, was successfully tested using a multimodal optical imaging approach (CLE, HSI, quantitative FA), which once again showed the good correlation of HSI with other optical imaging modalities and ischemia biomarkers [43].

Recently, HSI has been used in 22 human cases of patients undergoing esophageal resection to intraoperatively measure perfusion at the future anastomotic site [44]. HSI could successfully assess perfusion without interfering with the surgical workflow, indicating a possible correlation between low StO_2_ at the future anastomotic site and an increased probability for anastomotic breakdown (Figure 2B).

In a further study from the same group, the surgeons used HSI during 20 open pancreatic resections and could detect two cases of celiac trunk stenosis by using HSI intraoperatively [45]. Both of those clinical studies were aimed at exploring the usability of HSI intraoperatively during upper gastrointestinal surgical procedures. Larger studies are required to understand the role of HSI in preventing anastomotic complications during human cases of upper gastrointestinal surgeries.

### 5.3. Perfusion Assessment in Hepatopancreaticobiliary Surgery (HPB)

Pancreatic fistula (PF), namely the spillage of pancreatic fluid within the abdominal cavity, is the most frequent complication following pancreatic resection [84], negatively impacting overall surgical outcomes. Evidence indicates that PF is increased in the case of poor vascularization at the pancreatic stump [85]. However, currently pancreatic perfusion following resection is exclusively assessed by means of a subjective clinical judgment.

After the creation of a pancreatic partial ischemia model, researchers were able to quantify perfusion in porcine pancreas using a multimodal optical approach [46]. Among the techniques, HSI (with HYPER) was used, and it could successfully quantify pancreatic blood perfusion correlating well with ischemia biomarkers and other optical imaging techniques (such as FA and CLE). To our knowledge, this is the only existing study in which HSI has been used to assess pancreatic perfusion intraoperatively (Figure 2E).

In the field of hepatic surgery, HSI has been successfully used in a porcine hepatic ischemia model to differentiate total (hepatic artery and portal vein) inflow occlusion from partial (hepatic artery only) occlusion, using a methodologically sound setup [47]. The differentiation between isolated arterial or mixed (portal and arterial) ischemia has an important clinical value in the setting of liver transplant. In addition, for the first time a recent study showed that AI and HSI-based scores can predict liver viability during ischemic and reperfusion phases in a model of hepatic artery occlusion [48] (Figure 2G). In the future, if one can detect this kind of issue intraoperatively, it could well have a clinical impact on patients undergoing liver transplant and it might be helpful in the early diagnosis of graft thromboembolism.

Currently, during anatomical liver resection, parenchymal transection is performed subjectively, exclusively based on the visualization of the well-perfused versus ischemic parenchyma, which is particularly difficult in the case of bleeding or cirrhotic livers. To overcome this technical difficulty, some intraoperative guidance solutions involving near-infrared cameras have been proposed. The positive staining technique [86,87] and the negative staining technique [88,89,90] have been described in the past. The positive staining method is based on intraoperative ultrasound-guided ICG injection into the portal branches of the segments to be resected. As a result, their parenchyma will appear fluorescent. Negative staining involves a systemic intravenous injection after ligation of the Glissonian pedicles. In this way, the fluorescent signal diffuses to the whole liver, sparing the parenchyma of the segments to be resected. Both methods involve the use of an exogenous contrast agent. Differently, Urade et al. proposed a study in which HYPER served as an intraoperative navigation tool during anatomical hepatic resections in the porcine model [49]. After the ligation of selective vessels, it was possible to detect the parenchymal ischemia demarcation line with HSI acquisitions. The authors used a camera which can provide a perfusion cartography and this false-color image was overlaid onto the real-time video (Figure 2H). This could well help the surgeon to identify the parenchymal resection line and it served as guidance during parenchymal transection. However, as previously mentioned, currently HYPER remains an experimental tool, since several technical issues limit its immediate clinical translation.

### 5.4. Perfusion Assessment in Reconstructive Surgery

An excellent perfusion is a key factor for the correct healing of grafts such as flaps used in reconstructive surgery to cover up defects resulting from previous demolition surgical interventions. Recently, HSI has found an application in plastic surgery. In fact, in two clinical trials, the authors have used this technology to assess the viability of free and pedunculated flaps intraoperatively and in the postoperative phase [50,51]. In both studies, it was concluded that the perfusion variations displayed with HSI correlated well with the clinical behavior (failure or healing) of the grafts.

Both of the abovementioned studies are pilot trials exploring the capabilities of HSI as a tool which can assess perfusion of the flaps intraoperatively. However, also in this case, well-designed studies including a larger sample size are required to understand the real clinical value that intraoperative HSI might have during plastic surgery procedures.

### 5.5. Perfusion Assessment in Urology

Over the past years, several experimental [52,53] and clinical [54,55,56] studies using HSI to assess kidney perfusion during nephrectomies have been published. Those studies originating from the same group used a spectral scanning HSI system (specifically LCTF), which could analyze the reflectance spectra over a narrow portion of the EM spectrum (520 to 645 nm). However, this device could provide a quantification of the oxygenated hemoglobin over the analyzed area.

In proof-of-concept work using a porcine model, the authors successfully used their HSI camera to quantify renal perfusion during different vascular clamping maneuvers [52]. In a second set of experiments involving a larger number of pigs undergoing partial nephrectomy [53], researchers were able to quantify perfusion during gradual arterial inflow obstruction. Interestingly, the oxyhemoglobin percentage correlated with the arterial flow variations measured by means of an invasive ultrasound flow probe. In a further study, HSI was used to measure the remnant kidney perfusion in a small cohort of patients undergoing partial nephrectomies [54]. Successively, using the same HSI camera in 26 patients receiving partial nephrectomies, the authors noticed a correlation between high intraoperative baseline oxyhemoglobin and good postoperative renal function [55]. In a further study [56] in 37 patients undergoing partial nephrectomies, the authors did not notice any difference between artery-only and simultaneous artery/vein clamping in terms of oxyhemoglobin concentration measured with HSI.

Despite the interesting results shown in those studies, which support the use of HSI as a non-invasive reliable perfusion assessment method, there are consistent drawbacks which impaired the diffusion of this technology during urological cases. The issues are mainly related to the system used within those studies, namely a spectral scanning LCTF camera, which presented a relatively low spatial resolution and a long acquisition time (roughly 30 s).

In a recent study by Sucher et al. [57], researchers used a spatial scanning HSI camera to intraoperatively assess the perfusion of patients undergoing renal transplant. Although the patient cohort was relatively small (17 patients), the results were promising, showing a good correlation between a lower intraoperative perfusion and a delayed graft failure. Additionally, HSI proved useful to intraoperatively evaluate the ureteral viability, which is currently exclusively measured using a subjective visual assessment. Those remarkable results need to be confirmed on a larger scale. However, the intraoperative optical biopsy obtained with HSI could have an important clinical meaning, since it would provide an objective perfusion assessment during such a complex procedure such as a kidney transplant. Additionally, it would prevent from performing invasive biopsies on the graft, hence reducing the risk of potential related complications.

### 5.6. Perfusion Assessment in Neurosurgery

In an interesting paper combining an experimental and a clinical phase, a Japanese group used a modified spatial scanning HSI system to intraoperatively monitor brain perfusion in patients with Moya–Moya disease, undergoing a superficial temporal artery to middle the cerebral artery bypass [58]. First, the authors calibrated the perfusion algorithm that they set up within a small animal model. Successively, they monitored cerebral perfusion in vivo during clinical cases, demonstrating that after vascular anastomosis formation, HSI could detect a perfusion increase. The results matched with postoperative SPECT (single-photon emission computed tomography), which currently represents the gold standard in imaging to assess brain metabolism. To our knowledge, this was the only human case series in which HSI was successfully used to monitor the cerebral blood-flow intraoperatively. The remaining studies in which HSI is used to monitor brain oxygenation intraoperatively are single case reports in which the authors remarkably use custom-made LCTF [91] and snap-shot [92] HSI systems during surgical procedures for epilepsy.

## 6. Conclusions

Hyperspectral imaging is potentially a valuable tool for intraoperative guidance because it allows the performance of a non-destructive and contrast-free optical biopsy, analyzing the tissue chemical composition at a molecular level and virtually in real time.

Most of the preclinical research has been conducted on an HSI-based intraoperative perfusion assessment, which validated this kind of application in several organs mostly using sound experimental set-ups. Additionally, tissue oxygenation quantification is a well-known and easily accessible information to extract from the hypercube, which does not require the assistance of complex machine-learning algorithms. For this reason, nowadays this application represents the most practicable HSI use in the clinical practice, as confirmed by the existence of numerous clinical studies among diverse surgical disciplines.

On the other hand, the application of automatic tissue recognition of both anatomical structures and neoplastic tissue represents a more complex subject, although it would potentially have a tremendous clinical impact. However, it requires further multidisciplinary research to identify the spectral features required for each specific diagnostic query and to fully understand which tissue types can be reliably discriminated with HSI data. To reduce image acquisition and data processing times, only preselected target features of the spectrum will be imaged, that can be established with deep learning, and specific data extraction algorithms will be developed, which will ensure an extremely rapid information flow, resulting in a minimal time-lapse between image acquisition and information availability for the operating surgeon. HSI-based tissue recognition is still in the research and development stage. For this reason, its use outside research protocols still remains far from the routine clinical practice.

However, there are consistent drawbacks common for all intraoperative HSI applications, limiting the diffusion of this technology in the operating room. In fact, the available hyperspectral imaging systems are still relatively bulky, and are not suitable for minimally invasive surgery. While the acquisition time of most of the modern devices is now acceptable (roughly 6 s), they still do not provide information as high-quality videos and need consequently a relatively motionless subject to minimize motion artifacts.

However, the potential of this imaging modality in the surgical field has been understood and several research groups are focusing on modalities to overcome HSI limitations worldwide. As a result, soon, a miniaturized video HSI system combining perfusion quantification and specific automatic tissue recognition algorithms will be integrating part of the precision surgery operating room.

## Figures and Tables

**Table 1:** Schematization of HSI applications split into surgical subspecialties.

Application Category	Application/ Subcategory	Target	Subject (*n*)	Device Type	Acquisition Time	Spatial Resolution	Spectral Range	Reference
Tissue recognition	Cancer recognition	brain tumor	human (22)	Spatial scanning (two cameras)	40 + 80 s for both cameras	1004 × 1787 pixels	400 to 1700 nm	Fabelo H. et al. 2018 [24]
		brain tumor	human (16)	spatial scanning	~1 min	1004 × 1787 pixels	400 to 1000 nm	Fabelo H. et al. 2019 [25]
		brain tumor	human (16)	spatial scanning	ND	1004 × 1787 pixels	400 to 1000 nm	Martinez I. et al. 2019 [26]
		colorectal cancer	human (54)	spatial scanning	~6 s	640 × 480 pixels	500 to 1000 nm	Jansen-W., B et al. 2021 [27]
		colorectal cancer/esophageal cancer	human (22)	spatial scanning	~6 s	640 × 480 pixels	500 to 1000 nm	Collins T. et al. 2021 [28]
	Anatomical structures recognition	biliary structure	pig (3)	spectral scanning	~90 s	ND	650 to 1100 nm	Zuzak, K.J et al. 2008 [29]
		ureters, facial nerve	pig (3)	spectral scanning (two cameras)	ND	1392 × 1040 pixels and 640× 12 pixels	400–1100 nm and 850–1800 nm	Nouri, D et al. 2016 [30]
		artery, vein, bone, muscle, fat, connective tissue, parotid gland, and nerve	human (6)	spectral scanning	ND	1920 × 1080 pixels	380 to 1100 nm	Wisotzky, L. et al. 2018 [31]
		parathyroid, thyroid, and recurrent laryngeal nerve recognition	human (7)	spatial scanning	~6 s	640 × 480 pixels	500 to 1000 nm	Barberio, M. et al. 2018 [32]
		parathyroid, thyroid, and recurrent laryngeal nerve recognition	human (9)	spatial scanning	~6 s	640 × 480 pixels	500 to 1000 nm	Maktabi, M. et al. 2020 [33]
		artery, vein, nerve, muscle, fat, skin	pigs (8)	spatial scanning	~6 s	640 × 480 pixels	500 to 1000 nm	Barberio, M. et al. 2021 [34]
	Thermal ablation efficacy recognition	thermal effect monitoring during hepatic laser ablation	pig (1)	spatial scanning	~6 s	640×480 pixels	500 to 1000nm	De Landro, M et al. 2019 [35]
		thermal effect monitoring during hepatic laser ablation	pig (1)	spatial scanning	~6 s	640 × 480 pixels	50 to 1000 nm	De Landro, M et al. 2021 [36]
Perfusion assessment	Colorectal surgery	small bowel perfusion	pig (1)	spatial scanning (two devices)	ND	484 × 700 and 240 × 420 pixels	400–1000 and 900-1700 nm	Akbari, H. et al. 2010 [37]
		small bowel perfusion	pig (6)	spatial scanning	~6 s	640 × 480 pixels	500 to 1000 nm	Barberio, M. et al. 2019 [38]
		colonic perfusion	human (24)	spatial scanning	~6 s	640 × 480 pixels	500 to 1000 nm	Jansen-W., B et al. 2019 [39]
		colonic perfusion	human (32)	spatial scanning	~6 s	640 × 480 pixels	500 to 1000 nm	Jansen-W., B et al. 2021 [40]
		acute mesenteric ischemia	human (11)	spatial scanning	~6 s	640 × 480 pixels	500 to 1000 nm	Mehdorn, M. et al. 2020 [41]
	Upper-gastrointestinal surgery	gastric conduit perfusion	pig (5)	spatial scanning	~6 s	640 × 480 pixels	500 to 1000 nm	Barberio M. et al. 2020 [42]
		gastric conduit perfusion	pig (17)	spatial scanning	~6 s	640 × 480 pixels	500 to 1000 nm	Barberio M. et al. 2020 [43]
		gastric conduit perfusion	human (22)	spatial scanning	~6 s	640 × 480 pixels	500 to 1000 nm	Köhler, H. et al. 2019 [44]
		perfusion of upper abdominal organs	human (20)	spatial scanning	~6 s	640 × 480 pixels	500 to 1000 nm	Moulla, Y. et al. 2021 [45]
	Hepatopancreaticobiliary surgery	pancreatic perfusion	pig (6)	spatial scanning	~6 s	640 × 480 pixels	500 to 1000 nm	Wakabayashi, T, et al. 2021 [46]
		hepatic ischemia differentiation	pig (6)	spatial scanning	~6 s	640 × 480 pixels	500 to 1000 nm	Felli, E. et al. 2020 [47]
		hepatic ischemia/reperfusion injury	pig (5)	Spatial scanning	~6 s	640 × 480 pixels	500 to1000 nm	Felli, E. et al. 2021 [48]
		hepatic resection guidance	porcine (3)	spatial scanning	~6 s	640 × 480 pixels	500 to 1000 nm	Urade, T. et al. 2021 [49]
	Reconstructive surgery	flap perfusion	human (22)	spatial scanning	~6 s	640 × 480 pixels	500 to 1000 nm	Kohler, L.H. et al. 2021 [50]
		perfusion of free and pedicled flap	human (30)	spatial scanning	~6 s	640 × 480 pixels	500 to 1000 nm	Thiem, D.G et al. 2021 [51]
	Urology	renal perfusion	pig (7)	spectral scanning	<30 s	ND	520 to 645 nm	Tracy, C.R. et al. 2010 [52]
		renal perfusion (partial nephrectomies)	pig (14)	spectral scanning	<30 s	ND	520 to 645 nm	Best, S.L. et al. 2011 [53]
		renal perfusion (partial nephrectomies)	human (21)	spectral scanning	<30 s	ND	520 to 645 nm	Holzer, M.S. et al. 2011 [54]
		renal perfusion (partial nephrectomies)	human (26)	spectral scanning	<30 s	ND	520 to 645 nm	Best, S.L. et al. 2013 [55]
		renal perfusion (partial nephrectomies)	human (37)	spectral scanning	<30 s	ND	520 to 645 nm	Liu, Z.W. et al. 2013 [56]
		graft perfusion (kidney transplant)	human (17)	spatial scanning	~6 s	640 × 480 pixels	500 to 1000 nm	Sucher, R. et al. 2020 [57]
	Neurosurgery	brain perfusion	human (4)	ND	5–16 s	640 × 480 data points (pixels)	400 to 800 nm	Mori, M. et al. 2014 [58]

## Data Availability

The data presented in this study are available on request from the corresponding author.
